# Ecophysiological and Climatological Effects on Distribution of Vector Species and Malaria Incidence in India

**DOI:** 10.3390/ijerph9124704

**Published:** 2012-12-18

**Authors:** Takumi Kaga, Shunji Ohta

**Affiliations:** Department of Human Behavior and Environment Sciences, Faculty of Human Sciences, Waseda University, 2-579-15 Mikajima, Tokorozawa, Saitama 359-1192, Japan; E-Mail: izayoiduki-138@fuji.waseda.jp

**Keywords:** *Anopheles*, malaria incidence, coupled model, vector ecology, ecophysiology, climate factors

## Abstract

The magnitude of regional malaria risk is dependent primarily on the dynamics and distribution of the vector species, which are determined mainly by climate conditions. A coupled model with ecophysiological and climatological factors was developed to estimate the spatiotemporal distribution of the five species of dominant malaria vectors in monsoon Asia. Here, we examined how the potential distribution obtained from the model could explain trends in malaria incidence observed in India, which has the highest number of confirmed cases of malaria in Asia. Most notably, there was a significant positive correlation between annual malaria incidences and the maximum generation number of vectors for each state (*p* < 0.001). Malaria incidence tended to increase exponentially as vector generation number increased. In addition, the interannual variation in observed regional malaria incidences was synchronized with that of the potential number of vector generations. The observed seasonal peak of malaria incidences corresponded closely to the simulated appearance period of vector species, except for intensively irrigated areas that experience anthropogenic impacts on hydrologic conditions. Simulated vector distributions effectively expressed spatial and temporal prevalence of malaria in India. This novel approach to modeling based on vector ecology is an effective method for assessing malaria risk.

## 1. Introduction

India accounts for approximately two-thirds of all confirmed malaria cases in the South and East Asia regions. Interannual variation in the annual parasite index (API, malaria cases *per* 1,000 population *per* year), a commonly used index of the prevalence of malaria, has gradually decreased due to recent improvements in public hygiene, but there are large differences in total number of malaria cases and in the API between each state, reflecting the diverse topography, climate, and vector control programmes of India [1,2]. The API in most of India is less than 2, although the API ranges from 2 to 5 in scattered regions, and regions with API values higher than 5 are scattered in the states of Rajasthan, Gujarat, Karnataka, Goa, Madhya Pradesh, Chhattisgarh, Jharkhand, and Orissa, and eight states in the northeastern part of India [2,3,4]. Malaria cases in these areas account for more than 60% of cases in India as a whole [1,2]. 

Instead of the API, monthly parasite index values (MPI, malaria cases *per* 1,000 population *per* month) were used to analyze the seasonal prevalence of malaria on the basis of observational studies [5,6,7]. These studies suggest that MPI values are determined by the dynamics of vector species, as well as hygiene, distribution of the human population, and malaria parasites. In addition, there is a significant positive correlation between MPI and the density of *Anopheles stephensi*, the dominant mosquito vector of human malaria at the study site [5]. 

Several environmental factors, such as density-dependent processes and predation, play important roles in determining the distribution of *Anopheles* mosquitoes. In particular, climate factors have been established as being major determinants in the distribution of *Anopheles* mosquitoes, and consequently of malaria incidence [8,9,10,11]. Although relationships between malaria incidence and climate variables have been studied in many regions for malaria risk assessment [12,13,14], there is a paucity of literature on the relationship between vector species and climate variables. In the 1990s, most models used the methods of statistical and empirical approaches to estimate the geographical distribution of *Anopheles* mosquitoes [8,9,12]. Recently, the Malaria Atlas Project (MAP, www.map.ox.ac.uk) developed a new approach and generated maps for contemporary geographic distributions of all dominant mosquito vectors of human malaria globally in order to review the bionomics for each species in detail [15]. On the other hand, considering the effect of climate variables on the growth of malaria mosquito vectors, we have developed a new model coupled with the ecophysiological and climatological distribution of mosquito generations (ECD-mg), which can describe growth of *Anopheles* mosquitoes using simple climate factors [16]. The ECD-mg model successfully described the spatiotemporal distribution of *Anopheles* mosquitoes in monsoon Asia across cool-temperate to humid tropical regions [16]. The results obtained from the ECD-mg model predict the potential distribution of mosquitoes according to ecological and climatic factors, but additional variables, including human population, hygiene, and land-use change, contribute to determining the actual mosquito distribution. 

The main purpose of this study was to clarify the spatiotemporal relationship between the potential distribution of vector species and observed malaria incidences. First, the ECD-mg model and simple climate data were used to estimate the potential spatiotemporal distribution of *Anopheles* mosquitoes in India. Then, published observational data on temporal variation in malaria incidences were collected. Finally, we examined whether potential distributions of *Anopheles* obtained from the model could predict observed spatiotemporal trends in malaria in India. 

## 2. Methods and Data

### 2.1. Outline of the Coupled Model for Ecophysiological and Climatological Distribution of Mosquito Generation (ECD-mg)

**Figure 1:**
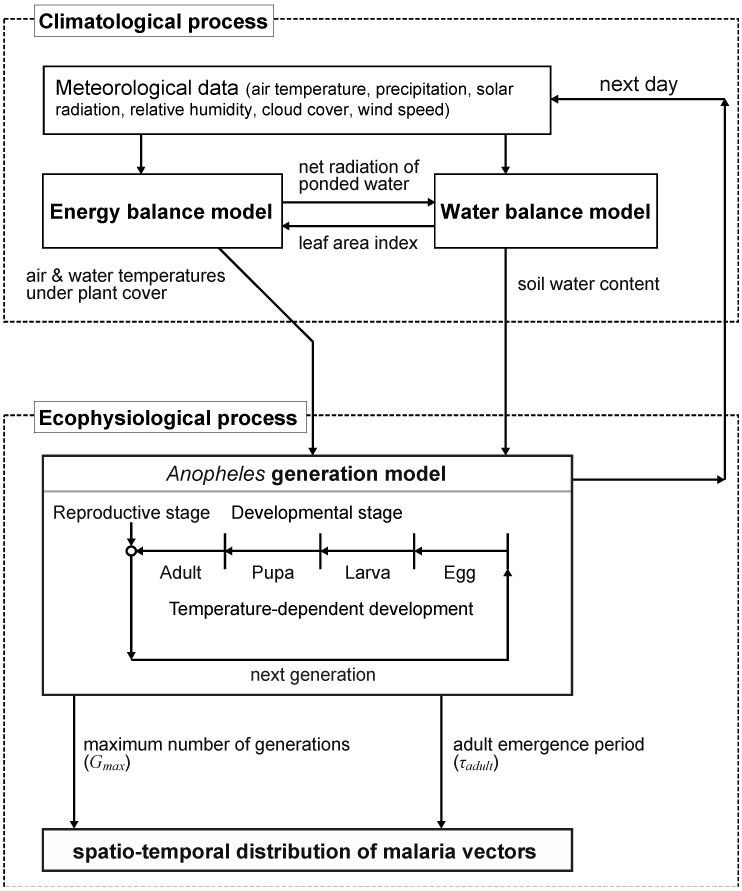
Flow diagram depicting how the ECD-mg model is used to simulate habitat suitable for immature and adult stages of *Anopheles* mosquitoes. Further details are provided in Ohta & Kaga [16].

The ECD-mg model consists of two processes: climatological and ecophysiological (Figure 1), with a 1-day calculation time interval. The climatological process estimates habitat conditions suitable for mosquitoes based on energy and water balance models, and the ecophysiological process incorporates these habitat conditions to simulate the temporal distribution of mosquitoes.

Records of the daily equilibrium between water temperature and soil water content are necessary in order to describe potential habitat for the immature stages of *Anopheles* [17,18]. However, there is no existing observational network for these data. Thus, the first stage of the ECD-mg model calculates water temperature and soil water content using energy and water balance models, and simple climate data, including air temperature, precipitation, solar radiation, cloud cover, relative humidity, and wind speed. It is critical that the model be precise in predicting these parameters for a natural, shallow-water, grassy habitats, which is a typical habitat of *Anopheles*. This type of habitat is suitable for *Anopheles culicifacies* and *Anopheles stephensi*, which are the most important malaria vectors in India [15,19].

When we estimated the temporal distribution of vector species for each representative site (as explained in the next section), air temperature, precipitation, and relative humidity data were obtained from the US National Oceanic and Atmospheric Administration (NOAA) [20] (except for the published precipitation data at one representative site [6]). Other climate data were taken from the surface climatology data of the Climate Research Unit (CRU, University of East Anglia) for the grid cell (1960–1990 climate normals and spatial resolution of 0.5° longitude × 0.5° latitude) [21]. To estimate the geographical distribution of vector species throughout India, all climate data were taken from the same CRU grid data [21]. Monthly values of climate data were converted to daily values using linear interpolation to ensure that the daily values were consistent with monthly averages or total values [16]. In addition, to calculate the soil water content, data for the plant-extractable water capacity of soil for each area (spatial resolution of 0.5° longitude × 0.5° latitude) were obtained from reference [22]. These data were determined in order to reflect the soil texture, organic content, and plant root depth or profile depth. 

Using habitat suitability data obtained as described above, the daily progression of mosquito growth was calculated by the model based on the mosquito life cycle (Figure 1). In the present model, this cycle was classified into four developmental stages (egg, larva, pupa, adult). The daily growth rate of the mosquitoes depended on the water or air temperatures for each stage. A new mosquito generation began on the day following a mosquito’s arrival at the reproductive adult stage, and the number of generations was cumulative. An adult emergence period (*τ_adult_*) was defined as the period in which adult mosquitoes can emerge. Emergence period directly affected the numbers of mosquito bites and malaria cases. The maximum number of generations (*G_max_*) was defined as the number of the last generation for a given year. Mosquito generation and adult emergence were summed over a 1-year period. In this model, the genus *Anopheles* represented all potential malarial mosquito species as a single entity.

### 2.2. Epidemiological Data, and Geographical Characteristics of Analysis Sites

Data from the National Vector Borne Disease Control Programme [4] on annual malarial cases for each Indian state were used to analyze spatial variation in occurrence of malaria from 2008 to 2010. These data were compiled for each state using unified methods as a whole of India. Human population data for each state derived from the Census of India [23] were used to calculate API values. The API values for each state from 2008 to 2010 were averaged to examine the recent trend in malaria cases. 

Published datasets of monthly malarial incidences were collected to analyze temporal variations in the disease. We examined locations with three or more years of recorded observations, and selected three datasets for analysis of MPI: Jodhpur (26°00′–27°37′N, 72°55′–73°52′E, 250–300 m above mean sea level: AMSL, Rajasthan state) [24], Dehradun (29°55′–30°32′N, 77°35′–78°20′E, 300–700 m AMSL, Uttaranchal state) [6], and Sundargarh (21º35′–22º35′N, 83º32′–85º22′E, 200–900 m AMSL, Orissa state) [7]. 

Jodhpur district is characterized by a tropical dry climate. Air temperature varies from 1 °C in winter to 49 °C in summer, and annual rainfall is approximately 300 mm [24]. Irrigated areas near the Indira Gandhi canal system are extensive in Jodhpur, creating conditions favorable for vector mosquito breeding, and it is thought that recurrence of a severe epidemic such as that of 1992 is likely [25]. Dehradun district is located in the northern part of India, and is characterized by a subtropical monsoon climate. Air temperature varies from 10 °C in January to 28 °C in May and June, and annual rainfall ranges from approximately 1,500–2,100 mm [20]. The majority of the rainfall in Dehradun is recorded between June and September during the southwest monsoon [6]. Sundargarh is located in a tropical humid climate, and forest covers more than half of the area in this district. The southwest monsoon provides rainfall between June and September, and the northeast monsoon provides rainfall in December and January, producing 1,600–2,000 mm of precipitation annually; temperature in Sundargarh ranges from 22–27 °C [7]. High rates of malaria morbidity and mortality in Sundargarh are due primarily to a prevalence of *falciparum* malaria in rural areas inhabited by tribal communities, which account for half of the population of Orissa state [7].

## 3. Results and Discussion

### 3.1. Spatial Relationship between Malaria Incidences and Potential Distribution of Vector Species

**Figure 2:**
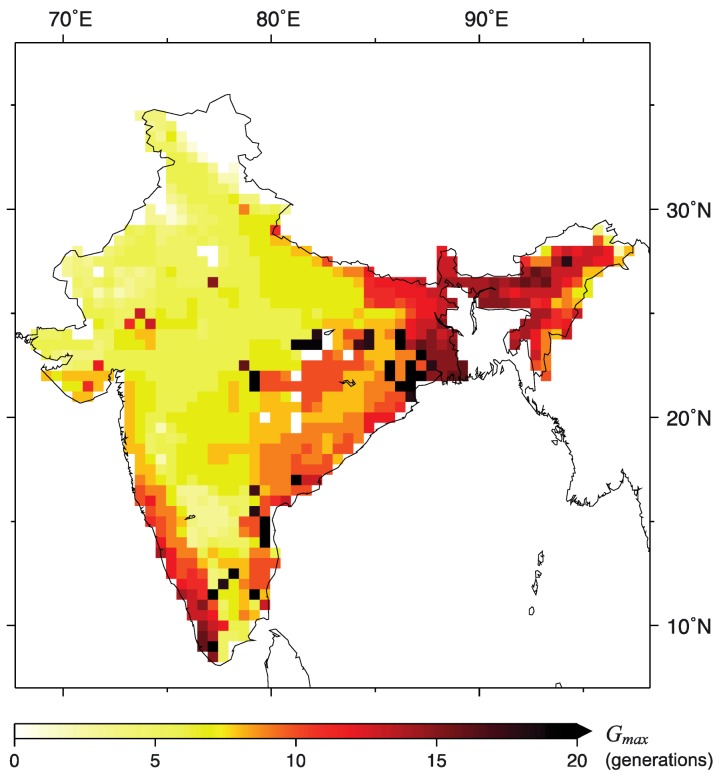
Geographical distribution of the maximum number of *Anopheles* generations (*G_max_*) produced annually.

*G_max_* varied from 0 to >20 (Figure 2), and its spatial distribution reflected variations in soil water content or water temperature. This result indicates *Anopheles* mosquitoes are unable to propagate in some parts of India, while in other areas mosquitoes reproduce year-round. Areas having a relatively high value of *G_max_* included the states of Orissa, West Bengal and Jharkhand, Kerala, and the northeastern Indian states, which produced more than 10 generations of *Anopheles* per year. Areas with relatively low *G_max_* values included parts of central and southern India, and the northwest, where Rajasthan state produced from 0 to seven generations. The results presented in Figure 2 reveal large differences in *G_max_*, depending on climate conditions. 

The ECD-mg model can predict the distribution using only simple climate data as input data. On the other hand, the MAP model [15] predicts the distribution based on environmental and climate variables, such as the normalized difference vegetation index (NDVI), the global land cover data (Globcover), and the current occurrence data of *Anopheles* mosquitoes. Although the input data are different depending on the models, the results of the spatial distributions of *Anopheles culicifacies* obtained from the ECD-mg model were mostly consistent with those obtained from the MAP work. However, the spatial distribution map of mosquitoes calculated with the MAP model has a higher resolution than the ECD-mg model due to the data format of the NDVI and Globcover. Although a lower spatial resolution of simple climate data is used to calculate the distribution with the ECD-mg model, our model can be applied easily to predict a temporal change in the future distribution of mosquitoes, since the required data are only simple climate data that can be easily applied to future climates. 

**Figure 3:**
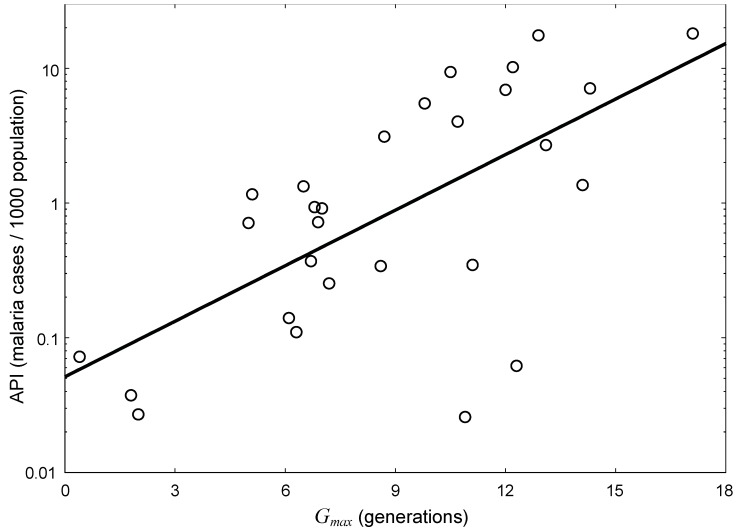
Relationship between averaged annual parasite index (API) from 2008 to 2010 and *G_max_* in each state (open circle) of India. The solid line indicates a regression line between API and *G_max_* (API = exp (0.316 *G_max_* − 2.970)).

State-averaged *G_max_* values were calculated from the spatial distribution of *G_max_*, for comparison with previously published average values of API [4,23]. Figure 3 denotes the relationship between API and the average values of *G_max_* for each state in India. A significant positive correlation was found between API and *G_max_* for each state (*r* = 0.61, *p* < 0.001), and incidences of malaria tended to increase exponentially as *G_max_* values increased (Figure 3). The number of generations per year is generally one of the most important parameters affecting the abundance of a species, and represents site suitability and climatic conditions [18,26,27]. Thus, it is likely that an increase in number or density of *Anopheles* mosquitoes would lead to an increase in malaria incidences.

However, another remarkable point from the results presented in Figure 3 is that there was an approximate 10-fold difference in API for a given *G_max_* value. This is partly because hygienic conditions in India vary greatly among states. Some states that fell above the regression line (Figure 3) are predominantly tribal districts, such as Chhattisgarh, Gujarat, Jharkhand, Madhya Pradesh, Maharashtra, Orissa, and Rajasthan [7,28]. In these states, socioeconomic and cultural factors play an important role in maintaining a high degree of malaria transmission [7,29]. In addition, the human population is distributed intensively in forested areas that are suitable for breeding of mosquitoes, particularly in Orissa state [7]. Conversely, Kerala and Bihar fell below the regression line (Figure 3), and are districts in which hygienic status has improved. In these districts, ambitious projects for malaria eradication have had a positive impact since the 1960s [30,31]. Furthermore, another suspected cause of the 10-fold difference is that the adult mosquito lifespan and the parasite development time were different for each state [10]. 

### 3.2. Temporal Relationship between Malaria Incidences and Potential Distribution of Vector Species

**Figure 4:**
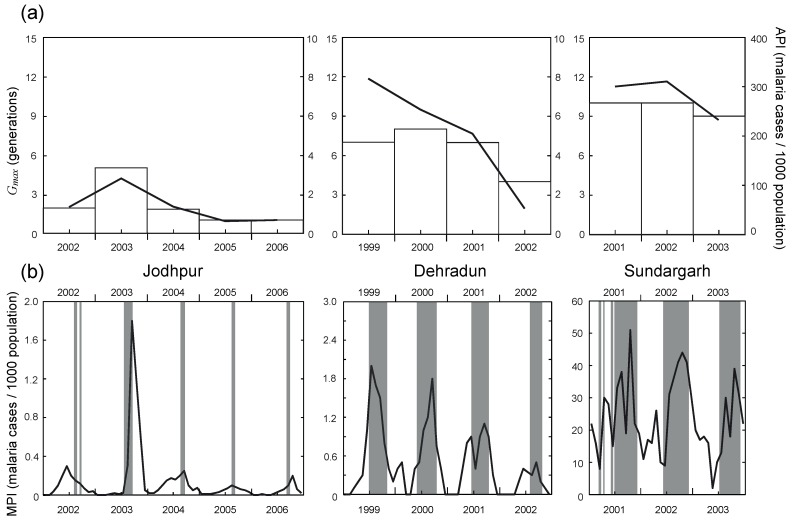
(**a**) Observed interannual variations in annual parasite index (API, solid line) and simulated maximum number of *Anopheles* generations (*G_max_*, histogram), and (**b**) Observed seasonal variations in monthly parasite index (MPI, solid line) and simulated adult emergence period (*τ_adult_*, solid gray bars) at three representative sites. Note that the solid gray bars do not represent the magnitude of adult emergence within these periods.

In order to verify whether actual malaria incidences could be explained by the temporal distribution of vector species simulated using the ECD-mg model, we extracted observational data for sites that were intensively examined in previously published research. Figure 4(a) compares interannual variation in observed malaria incidence with *G_max_* values obtained from the ECD-mg model for each site. The temporal variations in API were synchronized with those of *G_max_* at all sites, although the API in Sundargarh was 5 to 10 times higher than that in the other two locations due to poor hygienic conditions [7,29]. The cause of the interannual variation in *G_max_* was climate variability, because simple climate factors were the only input parameters in the ECD-mg model. The variation in *G_max_* led to differences in the abundance of mosquito vectors and incidences of malaria [18,26,27]. In particular, variability of precipitation affects soil moisture content, which in turn influences the survival and growth of immature stages of mosquitoes [14,17]. This suggests that climate conditions can alter occurrence rates of malaria by affecting vector species’ populations.

Figure 4(b) illustrates observed seasonal variations in malaria incidences and *τ_adult_* values obtained from the ECD-mg model for each site. The observed seasonal peak in MPI in Dehradun and Sundargarh corresponded closely to the *τ_adult_*. However, the simulated *τ_adult_* in Jodhpur was of shorter duration than the observed peak in MPI; the model produced *τ_adult_* values of approximately 1 to 2 months, which represents an underestimation for this region. Jodhpur contains many intensively irrigated areas, especially agricultural areas distributed in the northern part of the state [25]. Irrigation increases the amount of water available for the survival and growth of mosquitoes’ early life stages [25,33,34]. Consequently, this additional anthropogenic water could lengthen the active growing period for *Anopheles* spp., and it may be responsible for the significant lag of the major peaks in vector abundance [32,33]. The current ECD-mg model underestimated the *τ_adult_* in this intensively irrigated area because the model assumes natural climate conditions for the calculations with simple climate data. API in these irrigated areas was found by other researchers [24] to be considerably higher than in surrounding, unirrigated areas.

## 4. Conclusions

This study found a significant positive correlation between annual malaria incidences for each state in India and the maximum number of generations of *Anopheles* mosquitoes predicted by the ECD-mg model. Incidences of malaria tended to increase exponentially with increasing numbers of *Anopheles* generations. In addition, interannual variation in malaria occurrence at three intensively observed sites was synchronized with variability in number of *Anopheles* generations. Furthermore, the observed seasonal peak in malaria corresponded closely to the predicted adult emergence period of vector species, except for intensively irrigated areas, where anthropogenic activities impact regional hydrology. Vector distribution simulated with the ECD-mg model was able to express spatially and temporally the prevalence of malaria in India, where the dominant malaria vector species are *Anopheles culicifacies* and *Anopheles stephensi*. 

However, because the habitat of the dominant species in other countries is different from that in India, it is necessary to alter the assumption of the current ECD-mg model. Moreover, it is also necessary to consider the additional water, because supplying a large amount of irrigation water changed the distribution of the vector species and consequently altered the malaria risk. Another shortcoming of the model is that is not able to quantify the factors of seasonal vector abundance, parasite development time, and survival rate, which may affect seasonal malaria risk. There should be continued development of a population dynamics model that would be able to estimate the seasonal malaria risk more precisely [18,34]. 

The basic climate data (e.g., air temperature, precipitation, and solar radiation) required to adapt the ECD-mg model for use in other geographical areas are readily available. Therefore, this new approach, based on ecophysiological and climatological influences on vector species, shows promise as an effective method for estimating and projecting malaria risk under future climatic conditions. 
